# 2-(2*H*-1,3-Benzodioxol-5-yl)-1,3-benzo­thia­zole

**DOI:** 10.1107/S1600536812008914

**Published:** 2012-03-03

**Authors:** D. Lakshmanan, S. Murugavel, R. Selvakumar, M. Bakthadoss

**Affiliations:** a Department of Physics, C. Abdul Hakeem College of Engineering & Technology, Melvisharam, Vellore 632 509, India; b Department of Physics, Thanthai Periyar Government Institute of Technology, Vellore 632 002, India; c Department of Organic Chemistry, University of Madras, Maraimalai Campus, Chennai 600 025, India

## Abstract

In the title compound, C_14_H_9_O_2_S, the benzothia­zole unit is oriented at a dihedral angle of 7.1 (1)° with respect to the benzodioxole unit. The dioxole ring adopts flattened envelope conformation with the methyl­ene C atom at the flap. The crystal packing is stabilized by π–π inter­actions [centroid–centroid distances = 3.705 (1) and 3.752 (1) Å], C—H⋯π inter­actions and a short S⋯S contact of 3.485 (1) Å.

## Related literature

For background to the applications of benzothia­zoles in the chemical industry, see: Bradshaw *et al.* (2002 ▶); Delmas *et al.* (2002 ▶); Hutchinson *et al.* (2002 ▶). For the pharmacological activity of benzothia­zole derivatives, see: Repiĉ *et al.* (2001 ▶); Schwartz *et al.* (1992 ▶). For ring puckering analysis, see: Cremer & Pople (1975 ▶). For related structures, see: Baryala *et al.* (2010 ▶); Zhang *et al.* (2008 ▶).
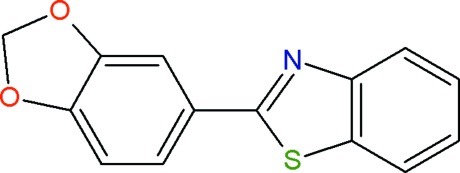


## Experimental

### 

#### Crystal data

C_14_H_9_NO_2_S
*M* *_r_* = 255.28
Orthorhombic, 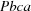

*a* = 6.3356 (2) Å
*b* = 16.3222 (5) Å
*c* = 22.0471 (7) Å
*V* = 2279.91 (12) Å^3^
*Z* = 8
Mo *K*α radiation
μ = 0.28 mm^−1^
*T* = 293 K
0.25 × 0.23 × 0.18 mm

#### Data collection

Bruker APEXII CCD diffractometer
Absorption correction: multi-scan (*SADABS*; Sheldrick, 1996 ▶) *T*_min_ = 0.934, *T*_max_ = 0.952
15338 measured reflections
3135 independent reflections
2243 reflections with *I* > 2σ(*I*)
*R*_int_ = 0.027

#### Refinement

*R*[*F* ^2^ > 2σ(*F* ^2^)] = 0.039
*wR*(*F* ^2^) = 0.105
*S* = 1.02
3135 reflections
163 parameters
H-atom parameters constrained
Δρ_max_ = 0.29 e Å^−3^
Δρ_min_ = −0.24 e Å^−3^

### 

Data collection: *APEX2* (Bruker, 2004 ▶); cell refinement: *APEX2* and *SAINT* (Bruker, 2004 ▶); data reduction: *SAINT* and *XPREP* (Bruker, 2004 ▶); program(s) used to solve structure: *SHELXS97* (Sheldrick, 2008 ▶); program(s) used to refine structure: *SHELXL97* (Sheldrick, 2008 ▶); molecular graphics: *ORTEP-3* (Farrugia, 1997 ▶); software used to prepare material for publication: *SHELXL97* and *PLATON* (Spek, 2009 ▶).

## Supplementary Material

Crystal structure: contains datablock(s) global, I. DOI: 10.1107/S1600536812008914/gk2459sup1.cif

Structure factors: contains datablock(s) I. DOI: 10.1107/S1600536812008914/gk2459Isup2.hkl

Supplementary material file. DOI: 10.1107/S1600536812008914/gk2459Isup3.cml

Additional supplementary materials:  crystallographic information; 3D view; checkCIF report

## Figures and Tables

**Table 1:** Hydrogen-bond geometry (Å, °) *Cg*1 is the centroid of the dioxole ring and *Cg*2 is the centroid of the C2–C7 benzene ring.

*D*—H⋯*A*	*D*—H	H⋯*A*	*D*⋯*A*	*D*—H⋯*A*
C5—H5⋯*Cg*1^i^	0.93	2.79	3.624 (2)	150
C14—H14*B*⋯*Cg*2^ii^	0.97	2.84	3.580 (2)	134

Symmetry codes: (i) 
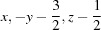
; (ii) 
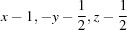
.

**Fig. 1.**
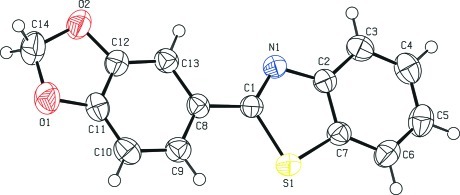
The molecular structure of the title compound with the atom numbering scheme. Displacement ellipsoids are drawn at the 50% probability level. H atoms are presented as a small spheres of arbitrary radius.

**Fig. 2.**
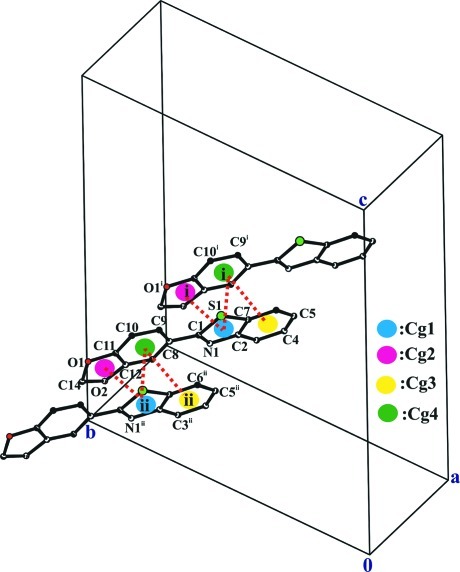
A view of the π···π interactions (dotted lines) in the crystal structure of the title compound. Cg1, Cg2, Cg3 and Cg4 are the centroids of the N1/S1/C1/C2/C7 thiazole ring, O1/O2/C11/C12/C14 dioxole ring, C2–C7 benzene ring and C8–C13 benzene ring, respectively [symmetry codes: (i)1 + x, y, z; (ii) -1 + x, y, z].

**Fig. 3.**
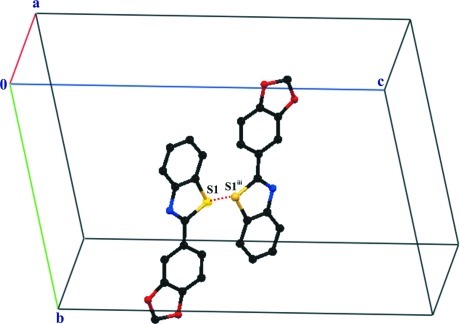
Part of the crystal structure showing a short S···S contact [symmetry code: (iii) = -x, 1 - y, 1 - z].

## supplementary crystallographic information

### Comment

Benzothiazoles are remarkable heterocyclic ring systems. They possess therapeutic value, are synthetic intermediates in the preparation of medicinal compounds and find numerous applications in chemical industry (Bradshaw *et al.*, 2002; Hutchinson *et al.*, 2002; Delmas *et al.*, 2002). Benzothiazole nucleus is associated with several pharmacological activities such as anti-tumor (Repiĉ *et al.*, 2001) and antimicrobial (Schwartz *et al.*, 1992). In view of this biological importance, the crystal structure of the title compound has been determined and the results are presented here.

Fig. 1. shows a displacement ellipsoid plot of (I), with the atom numbering scheme. The benzothiazole moiety is essentially planar [maximum deviation = -0.016 (1) Å for the C14 atom] and lies at an angle 7.1 (1)° with respect to the benzodioxole unit. The dioxole (O1/O2/C11/C12/C14) ring adopts an envelope conformation with the C14 (displacement = 0.03 (18) Å) atom as the flap atom and with puckering parameters (Cremer & Pople, 1975), q_2_ = 0.0882 (16) Å and φ_2_ = 143.8 (1)°. The geometric parameters of the title molecule agree well with those reported for similar structures (Baryala *et al.*, 2010; Zhang *et al.*, 2008).

The crystal packing is stabilized by π—π interactions with *Cg*3···*Cg*4^i^ and *Cg*1···*Cg*4^i^ seperations of 3.705 (1) Å and 3.752 (1) Å, respectively (Fig. 2; *Cg*1, *Cg*3 and *Cg*4 are the centroids of the N1/S1/C1/C2/C7 thiazole ring, C2–C7 benzene ring and C8–C13 benzene ring, respectively, symmetry code as in Fig. 2). The crystal packing (Fig. 3) is further stabilized by a short contact S1···S1^iii^ [3.485 (1) Å; symmetry code: (iii) = *-x, 1 - y, 1 - z*], which is shorter than the sum of the van der Waals radii of these atoms [3.60 Å].

### Experimental

A mixture of benzo[*d*][1,3]dioxole-5-carbaldehyde (0.15 g, 1 mmol), 2-aminobenzenethiol (0.125 g, 1 mmol), H_2_O_2_ (0.013 g, 0.4 mmol) and NH_4_Ce(NO_3_)_6_ (0.053 g, 0.1 mmol) was heated at 50°C for 12 h. After completion of the reaction, the reaction mixture was dissolved in EtOH and then poured into ice–water. The products were filtered, washed with ice-water, and subsequently dried. Recystallization of the product from ethyl acetate: hexanes (1: 10) yielded colourless crystals of the title compound (0.22 g; yield: 91%).

### Refinement

All the H atoms were positioned geometrically, with C–H = 0.93–0.97 Å and constrained to ride on their parent atom, with *U*_iso_(H) =1.5*U*_eq_ for methyl H atoms and 1.2*U*_eq_(C) for other H atoms.

### Crystal data

**Table:**

C_14_H_9_NO_2_S	*F*(000) = 1056
*M**_r_* = 255.28	*D*_x_ = 1.487 Mg m^−^^3^
Orthorhombic, *P**b**c**a*	Mo *K*α radiation, λ = 0.71073 Å
Hall symbol: -P 2ac 2ab	Cell parameters from 3185 reflections
*a* = 6.3356 (2) Å	θ = 2.7–29.5°
*b* = 16.3222 (5) Å	µ = 0.28 mm^−^^1^
*c* = 22.0471 (7) Å	*T* = 293 K
*V* = 2279.91 (12) Å^3^	Block, colourless
*Z* = 8	0.25 × 0.23 × 0.18 mm

### Data collection

**Table:**

Bruker APEXII CCD diffractometer	3135 independent reflections
Radiation source: fine-focus sealed tube	2243 reflections with *I* > 2σ(*I*)
Graphite monochromator	*R*_int_ = 0.027
Detector resolution: 10.0 pixels mm^-1^	θ_max_ = 29.5°, θ_min_ = 2.7°
ω scans	*h* = −7→8
Absorption correction: multi-scan (*SADABS*; Sheldrick, 1996)	*k* = −22→19
*T*_min_ = 0.934, *T*_max_ = 0.952	*l* = −30→30
15338 measured reflections	

### Refinement

**Table:**

Refinement on *F*^2^	Primary atom site location: structure-invariant direct methods
Least-squares matrix: full	Secondary atom site location: difference Fourier map
*R*[*F*^2^ > 2σ(*F*^2^)] = 0.039	Hydrogen site location: inferred from neighbouring sites
*wR*(*F*^2^) = 0.105	H-atom parameters constrained
*S* = 1.02	*w* = 1/[σ^2^(*F*_o_^2^) + (0.0459*P*)^2^ + 0.5885*P*] where *P* = (*F*_o_^2^ + 2*F*_c_^2^)/3
3135 reflections	(Δ/σ)_max_ < 0.001
163 parameters	Δρ_max_ = 0.29 e Å^−^^3^
0 restraints	Δρ_min_ = −0.24 e Å^−^^3^

### Special details

**Table:**

Geometry. All e.s.d.'s (except the e.s.d. in the dihedral angle between two l.s. planes) are estimated using the full covariance matrix. The cell e.s.d.'s are taken into account individually in the estimation of e.s.d.'s in distances, angles and torsion angles; correlations between e.s.d.'s in cell parameters are only used when they are defined by crystal symmetry. An approximate (isotropic) treatment of cell e.s.d.'s is used for estimating e.s.d.'s involving l.s. planes.
Refinement. Refinement of *F*^2^ against ALL reflections. The weighted *R*-factor *wR* and goodness of fit *S* are based on *F*^2^, conventional *R*-factors *R* are based on *F*, with *F* set to zero for negative *F*^2^. The threshold expression of *F*^2^ > σ(*F*^2^) is used only for calculating *R*-factors(gt) *etc*. and is not relevant to the choice of reflections for refinement. *R*-factors based on *F*^2^ are statistically about twice as large as those based on *F*, and *R*- factors based on ALL data will be even larger.

### Fractional atomic coordinates and isotropic or equivalent isotropic displacement parameters (Å^2^)

**Table:**

	*x*	*y*	*z*	*U*_iso_*/*U*_eq_	
C2	0.3453 (2)	0.55583 (8)	0.34631 (6)	0.0361 (3)	
C3	0.5016 (3)	0.53503 (10)	0.30472 (7)	0.0467 (4)	
H3	0.5044	0.5591	0.2665	0.056*	
C4	0.6519 (3)	0.47847 (11)	0.32101 (8)	0.0536 (4)	
H4	0.7554	0.4637	0.2932	0.064*	
C5	0.6523 (3)	0.44284 (10)	0.37826 (9)	0.0540 (4)	
H5	0.7562	0.4049	0.3882	0.065*	
C6	0.5011 (3)	0.46295 (10)	0.42024 (8)	0.0497 (4)	
H6	0.5013	0.4393	0.4586	0.060*	
C7	0.3482 (2)	0.51935 (9)	0.40395 (7)	0.0389 (3)	
C1	0.0600 (2)	0.61568 (8)	0.38384 (6)	0.0352 (3)	
C8	−0.1316 (2)	0.66515 (9)	0.38831 (6)	0.0361 (3)	
C9	−0.2413 (3)	0.67047 (10)	0.44268 (6)	0.0426 (4)	
H9	−0.1896	0.6427	0.4764	0.051*	
C10	−0.4255 (3)	0.71583 (10)	0.44857 (7)	0.0482 (4)	
H10	−0.4971	0.7194	0.4853	0.058*	
C11	−0.4962 (2)	0.75501 (9)	0.39779 (7)	0.0423 (3)	
C14	−0.6822 (3)	0.82343 (13)	0.32904 (9)	0.0610 (5)	
H14A	−0.8035	0.7967	0.3110	0.073*	
H14B	−0.6977	0.8821	0.3238	0.073*	
C12	−0.3898 (2)	0.74996 (9)	0.34317 (7)	0.0399 (3)	
C13	−0.2079 (2)	0.70649 (9)	0.33649 (7)	0.0395 (3)	
H13	−0.1373	0.7041	0.2996	0.047*	
N1	0.17928 (19)	0.61002 (7)	0.33610 (5)	0.0374 (3)	
O1	−0.6701 (2)	0.80397 (8)	0.39209 (6)	0.0589 (3)	
O2	−0.49343 (19)	0.79602 (7)	0.30038 (5)	0.0559 (3)	
S1	0.13899 (7)	0.55612 (3)	0.445920 (18)	0.05001 (14)	

### Atomic displacement parameters (Å^2^)

**Table:**

	*U*^11^	*U*^22^	*U*^33^	*U*^12^	*U*^13^	*U*^23^
C2	0.0354 (8)	0.0350 (7)	0.0380 (7)	−0.0045 (6)	−0.0022 (6)	−0.0013 (5)
C3	0.0491 (10)	0.0484 (9)	0.0426 (8)	−0.0004 (8)	0.0053 (7)	−0.0030 (6)
C4	0.0486 (10)	0.0508 (9)	0.0613 (11)	0.0041 (8)	0.0078 (8)	−0.0119 (8)
C5	0.0499 (10)	0.0412 (9)	0.0710 (12)	0.0092 (8)	−0.0066 (9)	−0.0042 (8)
C6	0.0515 (10)	0.0455 (8)	0.0523 (9)	0.0040 (8)	−0.0069 (8)	0.0062 (7)
C7	0.0387 (8)	0.0389 (7)	0.0391 (7)	−0.0033 (6)	−0.0013 (6)	0.0022 (6)
C1	0.0336 (7)	0.0396 (7)	0.0323 (6)	−0.0061 (6)	−0.0033 (6)	0.0018 (5)
C8	0.0326 (7)	0.0390 (7)	0.0367 (7)	−0.0043 (6)	−0.0021 (6)	−0.0033 (5)
C9	0.0443 (9)	0.0464 (8)	0.0372 (7)	−0.0018 (7)	0.0001 (7)	−0.0014 (6)
C10	0.0477 (9)	0.0531 (9)	0.0439 (8)	0.0007 (8)	0.0093 (7)	−0.0072 (7)
C11	0.0368 (8)	0.0372 (7)	0.0528 (8)	0.0005 (7)	0.0023 (7)	−0.0094 (6)
C14	0.0496 (11)	0.0653 (12)	0.0680 (12)	0.0174 (9)	−0.0045 (9)	−0.0032 (9)
C12	0.0391 (8)	0.0368 (7)	0.0439 (8)	−0.0016 (7)	−0.0050 (6)	−0.0027 (6)
C13	0.0381 (8)	0.0429 (8)	0.0375 (7)	−0.0013 (7)	0.0011 (6)	−0.0020 (6)
N1	0.0378 (7)	0.0409 (6)	0.0334 (6)	0.0007 (5)	−0.0006 (5)	0.0012 (5)
O1	0.0511 (8)	0.0612 (7)	0.0644 (8)	0.0187 (6)	0.0052 (6)	−0.0041 (6)
O2	0.0511 (7)	0.0618 (7)	0.0549 (7)	0.0179 (6)	−0.0032 (6)	0.0060 (5)
S1	0.0452 (3)	0.0662 (3)	0.0386 (2)	0.0075 (2)	0.00557 (17)	0.01540 (17)

### Geometric parameters (Å, º)

**Table:**

C2—C3	1.392 (2)	C8—C9	1.388 (2)
C2—N1	1.3926 (18)	C8—C13	1.412 (2)
C2—C7	1.403 (2)	C9—C10	1.388 (2)
C3—C4	1.374 (2)	C9—H9	0.9300
C3—H3	0.9300	C10—C11	1.365 (2)
C4—C5	1.390 (3)	C10—H10	0.9300
C4—H4	0.9300	C11—O1	1.3668 (19)
C5—C6	1.372 (3)	C11—C12	1.383 (2)
C5—H5	0.9300	C14—O2	1.424 (2)
C6—C7	1.384 (2)	C14—O1	1.428 (2)
C6—H6	0.9300	C14—H14A	0.9700
C7—S1	1.7243 (16)	C14—H14B	0.9700
C1—N1	1.2991 (18)	C12—C13	1.361 (2)
C1—C8	1.461 (2)	C12—O2	1.3731 (18)
C1—S1	1.7517 (14)	C13—H13	0.9300
			
C3—C2—N1	125.86 (13)	C10—C9—H9	118.8
C3—C2—C7	118.93 (14)	C8—C9—H9	118.8
N1—C2—C7	115.20 (13)	C11—C10—C9	116.70 (14)
C4—C3—C2	118.99 (15)	C11—C10—H10	121.7
C4—C3—H3	120.5	C9—C10—H10	121.7
C2—C3—H3	120.5	C10—C11—O1	127.88 (15)
C3—C4—C5	121.33 (16)	C10—C11—C12	121.77 (15)
C3—C4—H4	119.3	O1—C11—C12	110.34 (14)
C5—C4—H4	119.3	O2—C14—O1	108.49 (14)
C6—C5—C4	120.75 (16)	O2—C14—H14A	110.0
C6—C5—H5	119.6	O1—C14—H14A	110.0
C4—C5—H5	119.6	O2—C14—H14B	110.0
C5—C6—C7	118.21 (15)	O1—C14—H14B	110.0
C5—C6—H6	120.9	H14A—C14—H14B	108.4
C7—C6—H6	120.9	C13—C12—O2	128.00 (14)
C6—C7—C2	121.78 (15)	C13—C12—C11	122.54 (14)
C6—C7—S1	129.07 (12)	O2—C12—C11	109.44 (13)
C2—C7—S1	109.16 (11)	C12—C13—C8	116.84 (14)
N1—C1—C8	125.25 (13)	C12—C13—H13	121.6
N1—C1—S1	115.30 (11)	C8—C13—H13	121.6
C8—C1—S1	119.43 (10)	C1—N1—C2	110.70 (12)
C9—C8—C13	119.81 (14)	C11—O1—C14	105.23 (13)
C9—C8—C1	120.58 (13)	C12—O2—C14	105.57 (13)
C13—C8—C1	119.60 (13)	C7—S1—C1	89.62 (7)
C10—C9—C8	122.34 (14)		
			
N1—C2—C3—C4	−178.02 (14)	O1—C11—C12—C13	178.48 (13)
C7—C2—C3—C4	1.1 (2)	C10—C11—C12—O2	−179.03 (14)
C2—C3—C4—C5	−1.0 (3)	O1—C11—C12—O2	0.01 (18)
C3—C4—C5—C6	0.4 (3)	O2—C12—C13—C8	178.89 (14)
C4—C5—C6—C7	0.2 (3)	C11—C12—C13—C8	0.7 (2)
C5—C6—C7—C2	−0.1 (2)	C9—C8—C13—C12	−0.3 (2)
C5—C6—C7—S1	179.51 (13)	C1—C8—C13—C12	178.46 (13)
C3—C2—C7—C6	−0.5 (2)	C8—C1—N1—C2	−178.07 (12)
N1—C2—C7—C6	178.66 (14)	S1—C1—N1—C2	0.36 (15)
C3—C2—C7—S1	179.79 (12)	C3—C2—N1—C1	179.57 (14)
N1—C2—C7—S1	−1.03 (16)	C7—C2—N1—C1	0.45 (17)
N1—C1—C8—C9	−176.37 (14)	C10—C11—O1—C14	−175.20 (17)
S1—C1—C8—C9	5.26 (19)	C12—C11—O1—C14	5.84 (18)
N1—C1—C8—C13	4.9 (2)	O2—C14—O1—C11	−9.44 (19)
S1—C1—C8—C13	−173.44 (11)	C13—C12—O2—C14	175.75 (16)
C13—C8—C9—C10	−0.4 (2)	C11—C12—O2—C14	−5.88 (18)
C1—C8—C9—C10	−179.06 (14)	O1—C14—O2—C12	9.48 (19)
C8—C9—C10—C11	0.5 (2)	C6—C7—S1—C1	−178.68 (15)
C9—C10—C11—O1	−178.94 (14)	C2—C7—S1—C1	0.98 (11)
C9—C10—C11—C12	−0.1 (2)	N1—C1—S1—C7	−0.81 (12)
C10—C11—C12—C13	−0.6 (2)	C8—C1—S1—C7	177.71 (11)

### Hydrogen-bond geometry (Å, º)

Cg1 is the centroid of the dioxole ring and Cg2 is the centroid of the C2–C7 benzene ring.

**Table:**

*D*—H···*A*	*D*—H	H···*A*	*D*···*A*	*D*—H···*A*
C5—H5···*Cg*1^i^	0.93	2.79	3.624 (2)	150
C14—H14*B*···*Cg*2^ii^	0.97	2.84	3.580 (2)	134

Symmetry codes: (i) *x*, −*y*−3/2, *z*−1/2; (ii) *x*−1, −*y*−1/2, *z*−1/2.
